# Narrative review: Managing buprenorphine and opioid use disorder in the perioperative setting

**DOI:** 10.1111/papr.13427

**Published:** 2024-10-25

**Authors:** Lynn Kohan, Antje Barreveld, Sudheer Potru, Alaa Abd‐Elsayed, Eugene R. Viscusi

**Affiliations:** ^1^ Department of Anesthesiology University of Virginia Charlottesville Virginia USA; ^2^ Department of Anesthesiology and Perioperative Medicine Tufts University Boston Massachusetts USA; ^3^ Emory University School of Medicine Atlanta Georgia USA; ^4^ Department of Anesthesiology University of Wisconsin Madison Wisconsin USA; ^5^ Sidney Kimmel Medical College Thomas Jefferson University Philadelphia Pennsylvania USA

**Keywords:** acute pain service, analgesics, opioid, opioid use disorder, pain, postoperative

## Abstract

The opioid epidemic continues to have a staggering impact on millions of individuals and families across all socioeconomic levels and communities. Recent studies suggest high numbers of patients presenting for surgery with reported opioid misuse and/or opioid use disorder (OUD). Anesthesiologists often lack basic education to treat patients suffering with OUD or patients in recovery from this treatable disease. This manuscript will provide a review of the American Society of Anesthesiology and Pain Medicine Multisociety Working Group Practice Advisory recommendations on existing OUD treatment barriers and perioperative management best practices; it will also demonstrate the benefits that greater involvement of the anesthesiologist can have in managing patients with OUD perioperatively.

## INTRODUCTION

The opioid epidemic continues to devastate America. It is estimated that 6.7–7.6 million adults living in the US currently have an opioid use disorder (OUD).^1^ Previously, opioid overprescribing was considered the main driver for the escalating crisis: over 80 prescriptions were written per 100 people at the height of prescribing in 2012.^2^ However, despite a nearly 50% decrease in prescription opioids (to 43 prescriptions per 100 persons) in the United States,^2^ opioid overdoses still claimed the lives of over 100,000 Americans in the year ending in April 2021. These numbers sadly continue to escalate.^3^ Simultaneously, fear of prescribing opioids has led to a pain care crisis of sorts resulting in undertreatment of pain,^4^ abrupt cessation of chronic opioid therapy (with increased risk of illicit opioid use and even death from overdose or suicide), and decreased quality of life.^5^ The revised Centers Disease Control guidelines (2022)^6^ have reflected the need for a more nuanced and individualized use of prescription opioids.

The escalating number of lives lost and impacted by the opioid crisis is accompanied by escalating costs of healthcare. Healthcare costs for people who misuse opioids are approximately eight times higher than for those who do not. In 2020, the annual healthcare costs for these individuals exceeded $500 billion. In comparison, the entire Medicare and Medicaid programs cost $672.1 billion and $565.5 billion, respectively.^7^ Individuals who misuse opioids have higher rates of early termination of care and leaving the hospital with incomplete treatment.^8^ Spending on four federally illegal drugs (cannabis‐legal in some states, cocaine, heroin and methamphetamine) by Americans reached nearly $150 billion in 2016 and has increased considerably with the rising use of stimulants.^9^ Inadequate treatment of OUD leads to further morbidity, costly readmissions and mortality.^10^ Additionally, pre‐existing OUD has unique detrimental consequences during the perioperative period. Preoperative OUD has been associated with adverse postoperative outcomes in a variety of surgeries including total hip and knee arthroplasty,^11^ cesarean delivery,^12^ coronary artery bypass surgery,^13^ and spine surgery including prolonged length of stay, higher costs, and higher frequencies of surgical complications.^14^

## BACKGROUND

Anesthesiologists are leaders in perioperative medicine and pain management. Both undertreated pain and OUD have a significant impact on perioperative outcomes. Anesthesiologists have a key role in navigating the complex interplay of providing effective analgesia, decreasing risks associated with opioids, preventing the development of an OUD, and maintaining recovery. Recognizing risk factors in the development of OUD and having a basic understanding of the disease of addiction and barriers to treatment are essential for anesthesiologists to achieve these goals.

### Brief overview of OUD

The American Society of Addiction Medicine defines addiction as a treatable, chronic medical disease involving complex interactions amongst brain circuits, genetics, the environment, and an individual's life experiences. People with addiction use substances or engage in behaviors that become compulsive and continue despite harmful consequences.^15^ While addiction (better referred to as substance use disorder) is a medical disease that responds to appropriate treatment, the paths to treatment have historically remained outside standard and mainstream healthcare, thus stigmatizing treatment.

Given the prevalence of OUD, anesthesiologists should have a basic understanding of the disease state of OUD. OUD exists along a continuum from mild to severe.^16^, ^17^, ^18^, ^19^, ^20^, ^21^ The Diagnostic and Statistical Manual of Mental Health Disorders, 5th Edition (DSM‐V) defines OUD as a problematic pattern of opioid use leading to clinically significant impairment or distress (Table 1).^16^ The severity of the OUD is determined by the number of criteria met: mild 2–3, moderate 4–5, and severe >6. Hence, one may observe a spectrum of aberrant behavior related to opioid use in the perioperative setting.

**TABLE 1:** DSM‐5 OUD criteria.

American Psychiatric Association criteria for opioid use disorder^a^			
Impaired control	Social impairment	Risky use	Pharmacological criteria
1. Taking larger amounts or taking drugs over a longer period than intended	5. Problems fulfilling obligations at work, school or home	8. Using opioids in physically hazardous situations	10. Tolerance (i.e., need for increased amounts or diminished effect with continued use of the same amount)
2. Persistent desire or unsuccessful efforts to cut down or control opioid use	6. Continued opioid use despite having recurring social or interpersonal problems	9. Continued opioid use despite ongoing physical or psychological problems likely to have been caused or worsened by opioids	11. Experiencing withdrawal (opioid withdrawal syndrome) or taking opioids (or a closely related substance) to relieve or avoid withdrawal symptoms
3. Spending a great deal of time obtaining or using the opioid or recovering from its effects	7. Giving up or reducing activities because of opioid use		
4. Craving, or a strong desire or urge to use opioids			

*Note*: Criteria from American Psychiatric Association.^16^

^a^ Opioid use disorder can be classified by severity according to the number of criteria met: Mild (2–3 criteria); Moderate (4–5 criteria); Severe (6 or greater criteria).

The best treatment options are multidisciplinary, incorporating behavioral treatments (e.g., psychosocial counseling, contingency management, and others) with pharmacological therapies. There are currently three Federal Drug Agency‐approved medications for the treatment of OUD (MOUD): buprenorphine, methadone, and naltrexone. MOUD improves outcomes, retention in treatment, decreased morbidity and mortality. Still, recovery is usually a life‐long process that may require multiple attempts at treatment. Retention in treatment (for 6 months) may take an average of eight attempts, so encouragement and persistence are essential to survival and long‐term recovery.^17^

### Barriers to treatment

Despite the abundance of evidence for the effectiveness of MOUD, numerous barriers prevent access to treatment. A 2019 survey reported that less than 35% of adults with OUD had received treatment in the past year.^18^ Additionally, it has been estimated that there is a gap of 4–7 years between disease onset and initial treatment.^19^, ^20^ Barriers to treatment for OUD include stigma, inadequate education and training, delivery system fragmentation, regulatory and legal barriers, insurance coverage, and inadequate reimbursement that disincentivizes care.^21^

Stigma is a potent barrier to OUD treatment. Negative public attitudes towards individuals with OUD and other SUDs have been found to exceed those reported for other medical conditions, including mental illness.^22^ High rates of stigma have also been reported amongst health care professionals, leading to detrimental consequences for connecting patients with OUD treatment. Studies have found that primary care physicians' views, including measures of blame and desire to distance oneself towards patients with OUD were as high as the general public.^23^, ^24^, ^25^ Attention is increasing regarding the role of language in exacerbating stigma. Using non‐blaming terms, such as “person with substance use disorder”, has been found to decrease stigma versus language such as “substance abuser”.^26^, ^27^

Stigma also exists towards the use of MOUD, particularly methadone and buprenorphine, which may be viewed as substituting one drug of abuse for another.^28^ Indeed, there are significant disagreements about the value of these evidence‐based, life‐saving treatments even within the addiction community.^29^

Insufficient education and regulatory requirements also pose significant barriers to treatment access. SUD treatment is typically separated from mainstream healthcare, and education on OUD has neither been required nor standardized for clinicians in the United States.^21^ Addiction medicine is a young field that became a subspecialty of the American Board of Medical Specialties in 2015^30^ thus limiting clinicians' exposure. The separation from mainstream medicine further increases the inherent stigma described above, creating additional barriers to individuals with SUD seeking to obtain treatment. Unfortunately, restrictive regulations were previously established for treatment, requiring specialized prescribing clinics, observed dosing, and close behavioral monitoring, thus further isolating addiction treatment from primary/mainstream care and integrated treatment practices. These restrictions engendered feelings of isolation, hurt, shame, failure, mistrust, and low self‐esteem, adding barriers to treatment and increasing the stigma of addiction.^31^

We are optimistic, however, that the recent elimination of the X‐waiver in the U.S. via the Consolidated Appropriations Act of 2023^32^ can lead to drastic reductions in mortality, as was observed in France in 1996.^33^ The removal of this barrier will enable clinicians who encounter patients with OUD to treat them, even if they do not intend on building an addiction medicine practice themselves. These clinicians often are the front line, serving as the initial access point to treatment of OUD for vulnerable populations. Encouraging periodic prescribing may vastly expand access and even brief courses of buprenorphine initiated in the inpatient setting lead to decreased illegal opioid use.^34^ Increasing the comfort level of physicians to prescribe buprenorphine when clinically indicated and requested/agreed by the patient can be increased by utilization of free online tools from the Substance Abuse and Mental Health Services Administration (SAMHSA) as well as strengthening relationships with current OUD specialists.^35^ These bridges can enable physicians without substantial training or certification in addiction to initiate buprenorphine for vulnerable patients and refer to ongoing treatment. Anesthesiologists and pain physicians are prime clinicians who may fill this role and in doing so save lives.

## OUD AND THE ANESTHESIOLOGIST

Unfortunately, anesthesiologists and pain physicians may lack confidence and education on OUD treatment and prevention. OUD training has historically been limited in medical schools and residencies^36^ as well as how to treat comorbid pain in this patient population. Anesthesiologists may feel challenged with multiple barriers when treating both pain and addiction; indeed, one survey study of anesthesiologists and pain physicians demonstrates that the most‐perceived barriers to recommending starting buprenorphine for OUD included lack of appropriate clinical environment for prescribing, poor administrative support, poor payor mix, and the difficulty of treating OUD.^37^

It is understandable that some anesthesiologists, particularly those who only practice in the operating room setting, may feel uncomfortable initiating treatment for patients with OUD. The passage of the recent Medication Access and Training Education act, however, will require all clinicians (unless otherwise exempt due to previous training), seeking a DEA license, to obtain 8 h of training on management of patients with OUD.^38^

The purpose of this act according to SAMHSA is “Given the urgency of the nation's overdose crisis, the importance of practitioners receiving training in substance use disorders (SUD) cannot be overstated. Incorporating training on SUD into routine healthcare will enable practitioners to screen more widely for substance use disorders, treat pain appropriately, prevent substance misuse, and engage people in life‐saving interventions.”.^39^

Thus, this mandatory education hopes to provide necessary resources to increase all clinician's comfort level. Therefore, all anesthesiologists may play a role; the general anesthesiologist may screen and refer to treatment, while health systems with adequate resources such as an acute pain service can additionally initiate treatment and refer for ongoing care. Providing anesthesiologists with the education and tools needed to effectively care for perioperative patients with comorbid OUD in this manner has the potential to impact perioperative health outcomes and save lives, especially given the prevalence of the disorder.

Patients with OUD frequently present for surgery and may be misusing opioids. Nearly 12% of hospitalized patients have an active SUD.^40^ According to a 2022 study, as many as 2 in 5 patients in the preoperative period may present with unhealthy substance use, defined by the Tobacco, Alcohol, Prescription medications, and other Substance (TAPS) Tool,^41^ before elective surgery. Given the potential impact of substance use on surgical outcomes, increased recognition of the problem by screening patients is a critical next step for surgeons and perioperative care teams.^42^

Some patients may be in recovery and prescribed MOUD (buprenorphine, methadone, or naltrexone); however, many patients will present with active, untreated disease. Almost one‐third will terminate care early because of drug cravings, fear of mistreatment, financial and social pressures, and most commonly withdrawal.^7^ Patients with untreated OUD may have complicated hospital courses with decreased adherence to treatment plans, resulting in readmissions and poor outcomes.^7^ These scenarios present challenges and opportunities for anesthesiologists who are masters of the perioperative space.

While a formal diagnosis of OUD using the DSM‐5 criteria may require time and often repeated visits, anesthesiologists can screen for OUD by employing preoperative questionnaires using validated tools, such as the National Institute of Drug Abuse “Drug Abuse Screening Test” (DAST‐10) (Appendix 1),^43^ in a more time efficient manner.^43^ Thus, despite the complexities regarding OUD, recognizing and developing perioperative treatment plans for patients suffering with OUD does not have to be burdensome. If a patient screens positive, anesthesiologists can express their concern to the patient and/or notify the surgical team, medical team, and/or social worker to ensure appropriate treatment after surgery. The perioperative period is a particularly challenging time for patients with OUD.^44^ Chronic opioid exposure may lead to tolerance and hyperalgesia, potentially making effective postoperative analgesia with opioids more difficult.^45^ Multimodal analgesia and regional/ local anesthetic techniques are essential.

### Overview of perioperative care for patients with an OUD

#### Preoperative considerations

Early identification of patients with treated or untreated OUD is advantageous to allow time for the development of a coordination of care and patient education. The recent Health and Human Services Pain Management Interagency Best Practices Interagency Task Force concluded that preoperative SUD screening is recommended^46^ and use of more detailed assessment tools are recommended in patients who screen positive.^47^ Unfortunately, preoperative screening for opioid use is uncommon. A recent survey reported only 7% of patients were screened for preoperative opioid use. Perceived barriers to screening included insufficient time, logistics including clinic workflow, not perceiving screening as a priority, and lack of experience in chronic opioid use and OUD.^48^ Implementation of successful screening protocols have been reported despite these barriers including the EMPOWER consensus protocol for OUD screening and treatment stratification.^49^ The EMPOWER protocol identified not only those with OUD but stratifies patients according to severity of disease. More extensive screening such as the EMPOWER may be utilized prior to the day of surgery (including in the preoperative clinic) to help coordinate care.

Table 2 provides additional information to assess patients in the preoperative period.

**TABLE 2:** Overview of preoperative recommendations for patients with OUD.

Patients receiving MOUD	Patients with suspected or untreated OUD
Check prescription drug monitoring program (PDMP) Confirm MOUD dosing: Methadone dosing with methadone clinic Buprenorphine dosing with outpatient prescriber/PDMP Naltrexone dosing and formulation (oral vs. injectable [date]) with prescriber Plan MOUD preoperative dosing (see detailed discussion in text “Perioperative considerations for patients taking MOUD”): *Buprenorphine*: continue preoperative dose (consider BID or TID dosing) *Methadone*: continue preoperative dose *Naltrexone*: coordinate with prescriber, consider 48–72 h and oral bridge as indicated	Screen all patients for OUD NIDA quick screen DAST‐10 Consider use of “Screening, brief intervention, referral to treatment (SBIRT)”^a^ Assess for opioid misuse risk factors Opioid risk tool Assess for personal or family history of OUD, SUD, or untreated mental illness Consider obtaining urine drug screen^b^ Assess for signs of opioid withdrawal^54^, ^130^, ^131^ Nausea/vomiting Diarrhea Lacrimation Rhinorrhea Diaphoresis Shivering Piloerection Yawning Sneezing Restlessness Tremor Tachycardia
4 Set expectations and devise a patient‐centric and team‐based pain care plan Educate patients that some pain is expected and normal after surgery; however, every effort will be made to provide adequate analgesia Discuss non‐opioid, interventional, and non‐pharmacologic analgesic options Discuss post‐discharge pain care plans and post‐surgical opioid taper as indicated	5 Use non‐stigmatizing language (e.g., “person with OUD”) 6 Set expectations and devise a patient‐centric and team‐based care plan Consider recommending or prescribing MOUD Education patients that some pain is expected and normal after surgery; however, every effort will be made Discuss non‐opioid, interventional, and non‐pharmacologic analgesic options Discuss post‐discharge pain and OUD treatment plans

Abbreviations: DAST, Drug Abuse Screening Tool; NIDA, National Institutes on Drug Abuse; SBIRT, Screening, Brief Interventions, Referral to Treatment.

^a^ Evidence suggests growing evidence of efficacy in illicit drug misuse/abuse^132^ [60‐evidence supporting sbirt].

^b^ American Society of Anesthesiologists Task Force practice advisory for pre anesthesia evaluation does not comment on the preoperative use of UDS^133^ [61‐committee on standards and practice].

For patients receiving MOUD, a UDS along with documentation from your state's online controlled substance prescribing history (PDMP) can be considered preoperatively to confirm compliance with treatment. Care should be tailored to the individual patient's needs and may differ based on the type of MOUD the patient is receiving.

#### Perioperative considerations for patients taking MOUD

Table 3 provides a brief overview of management of patients on methadone and naltrexone, however, a robust discussion of these medications is beyond the scope of this manuscript.

**TABLE 3:** Review of food drug administration approved medications for MOUD.

	Methadone	Buprenorphine	Naltrexone
Mechanism of action	Full agonist at mu‐opioid receptor	Partial agonist at mu‐opioid receptor	Antagonist at mu‐opioid receptor
Dosing average range	80–100 mg daily	4–24 mg daily	380 mg IM depot injection 50 mg oral daily
Setting	Licensed outpatient treatment program (OTP)	Any medical setting	Any medical setting
Advantages	Care provided in highly structured supervised setting with built in resources Use in comorbid pain Low diversion	Care provided in a variety of outpatient models; less structured then outpatient Treatment program Lower risk of overdose Use in comorbid pain management Dosing flexibility	Low diversion Not an opioid Improved compliance No physical dependence Verifiable dosing Less stigma
Disadvantages	Caution with QTc prolongation Adverse effects: May increase LFTs, constipation and other opioid‐related side effects Caution with increased overdose risk Withdrawal with abrupt cessation	Diversion possible Adverse effects: Constipation and other opioid‐related side effects Withdrawal with abrupt cessation	Cannot be used for comorbid pain Adverse Effects: Flu like symptoms at first injection Requires ~10‐day opioid free period prior to initiation to avoid precipitated withdrawal

*Note*: Reprinted with permission from Barreveld et al.^50^

### Buprenorphine

Buprenorphine has unique pharmacological properties that may lead to confusion regarding its use in the perioperative period. Buprenorphine is a partial mu‐agonist and has the highest binding affinity for the mu‐receptor of readily available opioids while being a kappa‐ and delta‐receptor antagonist. Sufentanil alone has a higher binding affinity.^50^ Hence, buprenorphine can displace other opioids causing opioid withdrawal but can also treat opioid withdrawal symptoms in the absence of other opioids. Buprenorphine has several properties contributing to its efficacy in the treatment of OUD, including both mu (high affinity, low efficacy, and slow dissociation kinetics/long half‐life) and non‐mu‐opioid receptor factors (long terminal half‐life and lipophilicity).^51^ Kappa‐antagonism may be an important consideration in MOUD; most opioids have kappa‐ and delta‐agonism which lead to the euphoria/dysphoria and depression associated with full opioid agonists.^51^ The absence of kappa‐agonism and blocking of that receptor may interrupt the cycle of addiction and potentially decrease the risk of respiratory depression.^52^ Buprenorphine is commonly formulated with naloxone in a 4:1 ratio. The naloxone is inactive unless crushed or injected and used intransally or intravenously.^53^ Additionally, as a partial agonist at the mu‐receptor, buprenorphine is associated with decreased euphoria, gastrointestinal disturbances, and respiratory depression, leading to benefits such as decreased sedation and constipation, but enabling suppression of withdrawal and carvings.^51^ Despite its partial mu‐agonist property, it remains a potent analgesic, potentially via its interaction with other receptors such as the opioid receptor‐like 1.^51^ While the half‐life of buprenorphine is long (typically 24–42 h for transmucosal products), its analgesic half‐life is generally shorter (in the range of 6–8 h).^51^ Because of buprenorphine's high binding affinity, a relative dose‐dependent receptor occupancy can be established.^54^ Buprenorphine is Federal Drug Agency‐approved for both chronic pain (in a twice daily buccal film or weekly transdermal patch) as well as OUD (in an extended release parenteral subcutaneous injection, sublingual tablet, or film).^55^

Several investigators have quantified the dose‐dependent receptor occupancy of buprenorphine.^56^, ^57^ At 16 mg/day, perhaps 5%–10% of receptors remain available for other opioids. At 24–32 mg/day, virtually all receptors are occupied and additional buprenorphine or full agonists will provide little additional analgesia.^50^

## SUMMARY OF RECOMMENDATIONS FROM THE AMERICAN SOCIETY REGIONAL ANESTHESIA PAIN MEDICINE MULTI‐SOCIETY SUD WORKING GROUP

The output of the American Society Regional Anesthesia Pain Medicine SUD Multi‐Society Working Group created an education review and specific evidence‐based perioperative recommendations for anesthesiologists to guide intra‐ and postoperative management of patients with OUD.^58^ The two key conclusions were: (1) to decrease the risk of OUD recurrence, buprenorphine should NOT be routinely discontinued in the perioperative setting; and (2) buprenorphine can be initiated in untreated patients with OUD and acute pain in the perioperative setting to decrease the risk of opioid recurrence and death from overdose. We will next provide a review of these 2 recommendations.

### Perioperative management of patient on buprenorphine

In the past, there has been debate about whether to continue buprenorphine perioperatively because of confusion about the analgesic efficacy of this drug in the setting of profound opioid tolerance and opioid binding potency. Given this complexity recommendations varied widely. Previous recommendations often recommended discontinuing buprenorphine for at least 5 days priors to surgery.^59^, ^60^, ^61^, ^62^ There is further discrepancy in health system protocols in regards to the time to hold buprenorphine prior to surgery. Boston Medical Center recommended holding buprenorphine for 1 day prior to surgery.^63^, ^64^ On the other hand, guidelines from the University of Kentucky recommended to continue buprenorphine.^64^ Furthermore, the Veterans Health Administration had 2 recommendations; one in which buprenorphine is discontinued and another in which it was continued, although they noted it should be continued in most patients.^64^ Despite this suggestion, a recent survey conducted in a large Veterans's health care system reported 66% of patients experienced a buprenorphine hold at some point perioperatively.^65^

There is growing consensus, however, that buprenorphine should be continued in the perioperative period. MacIntyre et al.^66^ demonstrated that pain relief and opioid requirements in the first 24 h after surgery in patients receiving buprenorphine or methadone MOUD had no difference in pain intensity ratings whether they continued or discontinued treatment during the first 24‐h postoperatively. Those that discontinued treatment consumed large quantities of IV‐PCA opioids. Additionally, a recent retrospective review of 275 patients found that patients continued on buprenorphine utilized less morphine milliequivalents at all time periods (PACU, PACU discharge, 24 h, 24–48 h postoperatively) than patients who discontinued their buprenorphine. Patients who were continued on their buprenorphine were also less likely to require inpatient pain consults.^67^ Several other studies also reported adequate postoperative pain control in patients taking Buprenorphine.^66^, ^68^, ^69^, ^70^, ^71^

Discontinuing buprenorphine perioperatively was associated with decreased rates of returning to treatment. Wyse et al.^65^ reported that amongst patients who had buprenorphine discontinue perioperatively, 13% of patients had not restarted within 30 days of surgery, and these incidences increased to 25% and 33% at 6 and 12 months postsurgery, respectively. Reasons for lack of buprenorphine resumption included delay in appointment with their buprenorphine prescriber and/or resuming the prescription postsurgery, lengthy preoperative hold of buprenorphine, initiate an opioid for postsurgical pain without concomitant buprenorphine and returned to use.^65^ SAMHSA recommends that if buprenorphine was held prior to surgery, attempts should be made to reinitiate it prior to hospital discharge.^72^ Discontinuing buprenorphine is particularly alarming given that discontinuation has been associated with an unacceptably high incidence of disease recurrence, return to illicit drug use, and overdose.^22^, ^73^, ^74^, ^75^, ^76^ The risk of overdose is particularly high in the immediate period following buprenorphine cessation,^22^ thus perioperative cessation may endanger patients.

As the risks for discontinuing buprenorphine become more evident, recommendations to continue buprenorphine are becoming more common.^19^, ^64^, ^74^, ^77^, ^78^ Mass General Hospital recently changed their recommendations suggesting instead of discontinuing buprenorphine that buprenorphine should be continued in patients on 16 mg or less and when pain is expected to be mild. For doses >16 mg and pain is expected to be moderate or severe, they recommended to split the dose to enable more effective analgesia and to reduce the dose on the day prior or day of surgery to enable effective analgesia with full mu‐agonists.^64^ These newer recommendations incorporate the findings that adequate analgesia can be achieved in patients on buprenorphine as well as the risks of overdose of return to use when buprenorphine maintenance is discontinued.^79^

While there is now general agreement that buprenorphine should be continued perioperatively regardless of type of surgery,^45^, ^71^, ^80^ there is less consensus on the need to taper.^81^, ^82^ There are increasing recommendations to continue home dose of buprenorphine. Baresh et al.^81^ recently performed a literature review and recommended continuation of buprenorphine at home dose. Kohan et al.^19^ also recommended continuing the home dose of buprenorphine. Additionally, a recent practice advisory by Goel et al.^83^ suggested the continuation of the home buprenorphine dose in most cases. It is important to consider risk factors for OUD recurrence prior to considering tapering the patients' home buprenorphine dose.^73^ Risk factors include <20 months of treatment with buprenorphine, a positive UDS within the last 20 months, discharge from the hospital without maintenance of buprenorphine, and lack of communication with the patient's buprenorphine prescriber.^83^ Other authors have recommended maintaining but decreasing the dose of preoperative buprenorphine particularly when the expected postoperative pain is expected to be of moderate to severe intensity.^59^, ^60^, ^61^ Lembke et al.^84^ recommended decreasing the dose of buprenorphine to 12 mg 2–3 days prior to surgery, with return to maintenance dose by 3 days postsurgery. Quaye and Zhang,^78^ recently recommended continuing home dose for minor surgeries but decreasing the dose to 8 mg daily for major surgeries.

Similar to patients on preoperative opioid therapy, patients with OUD often have developed physiologic dependence to opioids. This opioid tolerance, whether from the use of illicit opioids or from MOUD should be considered when dividing an adequate analgesic plan. Additionally, patients do not derive sustained analgesia from maintenance doses of MOUD. The analgesic duration of action of methadone and buprenorphine is 4–8 h, shorter than the duration of action to reduce cravings.^85^, ^86^, ^87^, ^88^ Clinicians may need to administer higher doses or more potent opioid analgesics than would be provided to opioid naïve patients.^77^ In contrast, Roberts and Meyer‐Witting^89^ recommends increasing the buprenorphine dose by 25% for major surgeries.

It is important to utilize a multimodal analgesic regimen including non‐pharmacologic treatments when caring for patients on buprenorphine for OUD.^90^ Adequate treatment of pain is essential. Literature suggests that undertreating acute pain can lead to decreased responsiveness to opioid analgesics, thus making subsequent pain more difficult to treat.^91^, ^92^ Administration of NSAIDS and acetaminophen, adjuvant analgesics such as tricyclic antidepressants or anticonvulsants should be co‐administered accordingly.^93^, ^94^, ^95^ Comprehensive recommendations for the use of a multimodal analgesic regimen were reported in the Multisociety Guidelines by Kohan et al.^19^

**FIGURE 1:**
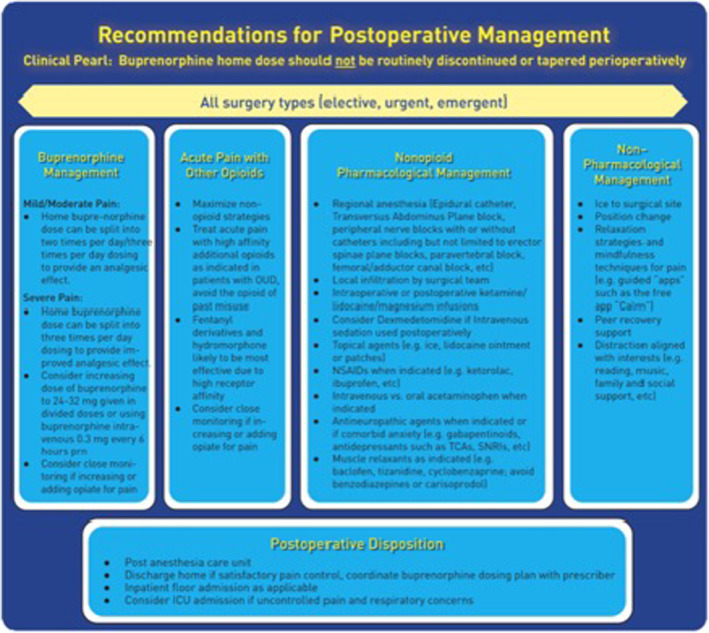
Multimodal recommendations for perioperative management. Reprinted with permission from Kohan et al.^58^

Figure 1 multimodal analgesia recommendations.^19^

If full mu‐agonists are deemed necessary to treat postoperative pain, opioids with high receptor binding affinity (sufentanil, fentanyl, and hydromorphone) are recommended.^55^ Sufentanil has the highest intrinsic efficacy of our available full opioid agonists, reaching maximal analgesic effectiveness at only 50% receptor occupancy. Hence, small amounts of sufentanil will provide the most effective analgesic benefit.^51^ Short acting full mu‐agonists can be titrated to effect using the lowest dose necessary.^95^ While concerns may exist that additional opioid analgesics in patients receiving MOUD will result in respiratory or central nervous system depression, this phenomena has never been clinically demonstrated.^63^ Clinicians may also have concerns that exposing patients to opioids may increase the risk of return to use; however, there is no evidence that exposure to opioids analgesics in the presence of acute pain increases the risk of return to use in patients receiving MOUD.^63^ In fact, untreated pain has been found to be a trigger for return to use.^96^ If full mu‐agonists are needed, patients should be provided the lowest effective dose or shortest period of time.^45^

Additionally, the dose of buprenorphine can be divided to enhance the analgesic properties of the medication^77^, ^86^ since as previously noted the analgesic half‐life is about 4–8 h.^85^

Discharge planning should include communication with the patient's buprenorphine prescriber (a “warm hand‐off”).^45^ A detailed plan to taper full mu‐agonists is essential.^19^ Strategies to maximize return to use should be incorporated.^45^ If the patient is at high risk of recurrence and in need of full mu‐agonists for sufficient analgesia, consider providing short 1–3 day scripts to avoid overuse. Buprenorphine is a powerful analgesic and addition of standard full opioid agonists may not be necessary with concomitant utilization of multimodal analgesics. While the analgesic ceiling of buprenorphine has not been clearly elucidated, short‐term supplemental buprenorphine may be sufficient to aid analgesia in patients on low to moderate buprenorphine doses.^97^

## BUPRENORPHINE PERIOPERATIVE MOUD CONSIDERATIONS

- Continue home dose.
- Do not routinely reduce and never discontinue preoperatively.
- May divide into BID or TID dosing for improved analgesia.

Coordinate MOUD and postoperative pain care plan with surgeon, patient, and outpatient buprenorphine prescriber.

### Discharge planning

It is important to support patients with active OUD or in recovery during the perioperative and in particular postoperative period for pain, opioid withdrawal, or aberrant opioid use. The postoperative plan should be tailored to meet the patient's individual needs as determined by analgesic requirements, surgical complexity, opioid use history, medical comorbidities, support systems, and overdose risk. Additionally, all decision‐making regarding management and use of controlled substances should be shared with the patient, the surgical team, primary care team, and the outpatient addiction medicine clinician or MOUD prescriber.^45^ If needed, a short course of a full mu‐agonist can be prescribed concomitantly with the patient's home dose of methadone or buprenorphine. Tapering instructions, to wean off of the full mu‐agonist, should be provided. The patient should also be educated on the use of harm reduction strategies and availability of naloxone prior to discharge as described below.^19^, ^45^

Additional discharge recommendations include emphasis on overdose and infection prevention, as well as linkage to care.^98^

### Recommendations on initiating buprenorphine in patients with untreated OUD

As the opioid epidemic continues, there is a growing need to increase access to care at all health care access points. Expanded access to MOUD reduces the number of overdose deaths.^4^, ^22^ The period of hospitalization has been found to a particularly vulnerable time for patients with OUD. The most compelling reason to consider initiating treatment prior to hospital discharge is the unacceptably high risk of death of a patient with OUD in the first 28 days following a hospitalization; this risk may be higher than any other time in these individuals' lives.^10^ There is thus a reachable moment for anesthesiologists/pain physicians to assist in preventing death by considering initiation of MOUD, particularly with buprenorphine. Hospitalization provides a unique opportunity to establish trust, treat pain and opioid withdrawal, and coordinate outpatient follow‐up treatment with the help of coordinated care teams. Since not all hospitals have the resources to engage in high‐quality coordinated care, it should be noted that evidence suggests that Buprenorphine is life‐saving, even if only taken for a short period.^99^, ^100^ The number needed to treat with buprenorphine is less than three.^101^, ^102^ Buprenorphine treatment was also associated with a 37% reduction in all‐cause mortality during the year after a nonfatal overdose.^4^, ^103^

Additional reasons for expanding access to MOUD during hospitalization include patient interest. Patients have been found to be interested in treatment for OUD during a hospital admission for a drug‐related catastrophic illness.^104^ Patients may be ready for change for many reasons including the fear of poor healthcare outcomes (loss of limbs, death), the absence of triggers and forced abstinence from illegal street drugs, and reconnections with family and previous lives. Also, they may trust their care team who has treated them with respect and kindness. In surveys, 67% of hospitalized people who use drugs state that they would like to cut back or quit, 44% of people with OUD report a strong interest in MOUD.^105^

Patients with OUD are more likely to be hospitalized than patients without OUD.^106^ Hence, despite perceived challenges that might arise from the prescriber side in initiating buprenorphine in the perioperative period, opportunities exist for patients to be initiated and managed with buprenorphine particularly through the expertise of an acute pain service.^107^

### Buprenorphine initiation evidence

There is increasing evidence that MOUD can be safely initiated in hospitalized and acute care patients.^39^, ^41^, ^108^, ^109^, ^110^, ^111^ In a randomized trial Liebshultz et al.^39^ reported a decreased incidence of illicit opioid use and increase retention in treatment over a 6 month period compared with detoxification in patients with OUD. Shanahan et al.^109^ reported an 82% follow‐up rate at an outpatient methadone program in hospitalized patients started on methadone. Microdosing induction techniques have also been successfully implemented in hospitalized patients.^112^, ^113^ While traditional microdosing techniques may take longer to achieve adequate analgesia and reduction of cravings, a recent case series suggests evidence of successful rapid microdosing technique in hospitalized patients.^114^ Adequate postoperative analgesia was achieved without precipitating withdrawal in both cases with full mu‐agonists being discontinued within 3–5 days.^114^ An additional case study reported successful rapid microinduction using IV buprenorphine in one patient requiring postoperative analgesia and another previously on methadone.^115^

Furthermore, there is a large body of evidence in the emergency medicine field reporting successful initiation of buprenorphine.^116^, ^117^, ^118^, ^119^ Synder et al. reported successful initiation of high dose buprenorphine including patients with fentanyl use. Overall almost 90% of patients were initiated using high dose (8–32 mg buprenorphine) with an incidence of 1.6% of precipitate withdrawal. No cases of precipitated withdrawal had to be hospitalized.^120^ Additional studies from the emergency medicine literature suggest that clinicians without prior specialized training in addiction medicine increased first time prescribing of buprenorphine after a brief 30 minute education intervention.^121^ The authors concluded that a brief educational intervention could be used to achieve first time prescribing, and improve knowledge around buprenorphine and opioid withdrawal.^121^

In addition, buprenorphine has been successfully initiated in the pre‐hospital setting by paramedics.^122^, ^123^

These studies suggest that buprenorphine can be initiated by non‐addiction medicine specialists during times of acute care. Emergency medicine clinicians, similar to most anesthesiologists, provide acute point of contact care and are not typically engaged in long‐term patient follow‐up. The emergency medicine literature, suggests, however, successful initiation of buprenorphine and hand‐off of care. Thus, strategies to adopt similar approaches within the perioperative setting can be considered. While large scale studies are needed, buprenorphine initiation has been accomplished with success in the postoperative period typically following all surgical procedures, while still providing adequate pain control and stabilization on standard opioids.^107^, ^124^ Buprenorphine was successfully initiated by the acute pain service in a patient with refractory spasms secondary to C4 tetraplegia.^124^ Additionally, Patel et al.^107^ reported successful initiation of buprenorphine by the acute pain service for comorbid OUD and acute pain in 7 patients; with 5/7 patients filling at least one buprenorphine script in 30 days post admission.

**FIGURE 2:**
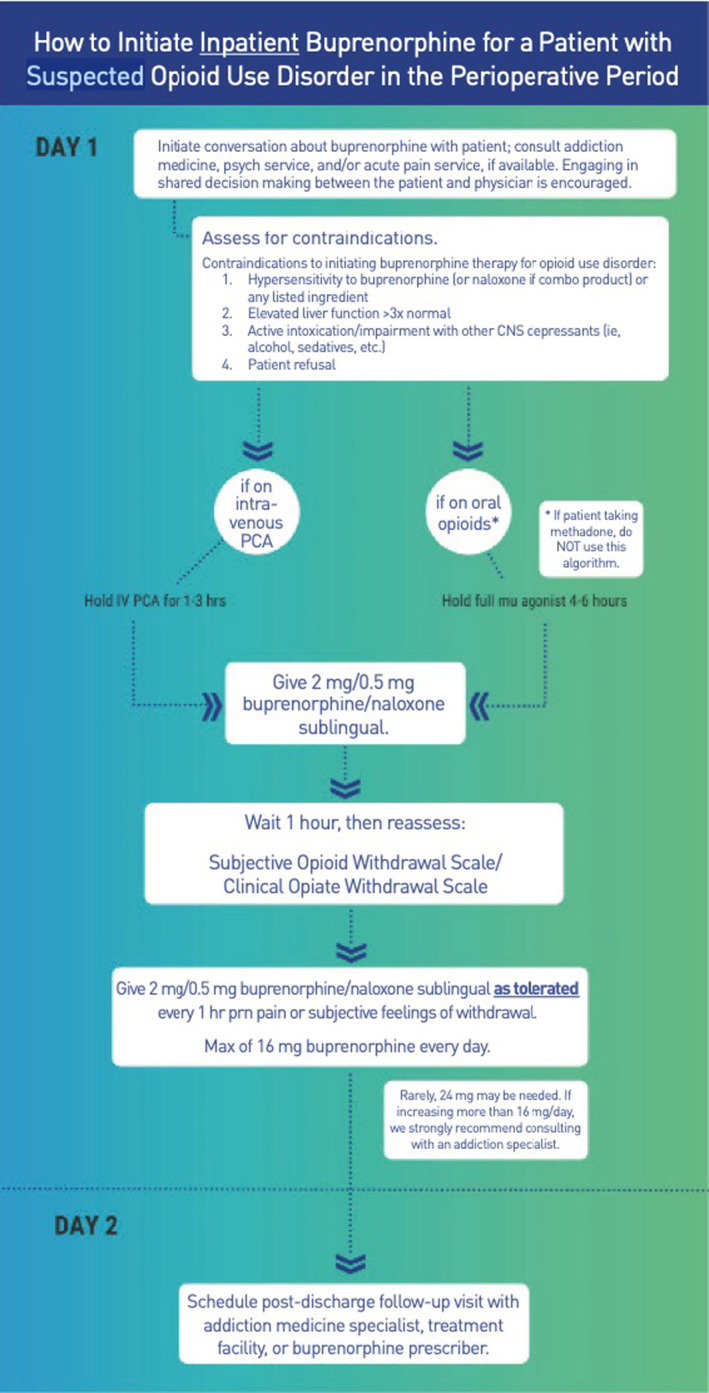
How to initiate Buprenorphine in the hospitalized postoperative patient. How to initiate buprenorphine for a patient with suspected opioid use disorder (OUD) in the perioperative period. *We do not recommend using this algorithm (eg, initiating buprenorphine) in patients with chronic pain who are currently being prescribed long‐acting opioids in the perioperative period. Clinical Opioid Withdrawal Scale (COWS). Subjective Opioid Withdrawal Scale (SOWS). CNS, central nervous system; PCA, patient‐controlled opioid analgesia. Reprinted with permission from Kohan et al.^58^

In order to successfully initiate buprenorphine, patients must first be agreeable with treatment and expectations must be managed before induction. Different protocols can be utilized to initiate buprenorphine in hospitalized patients. Figure 2 details an inpatient buprenorphine induction utilized by the team of authors (COWS Appendix 2).^125^

While clinicians may choose to adopt various induction protocols, evidence to support some form of buprenorphine induction is supported by reports that patients who do not start treatment are less likely to follow‐up with treatment on their own.^41^

It most assuredly takes time, attention, and a desire to help these challenging patients, but given the current state of reduced access to care and evidence of reduced death with treatment, initiation of treatment in this setting is a viable option.

As the opioid crisis escalates and more patients enter the perioperative space already receiving buprenorphine MOUD, anesthesiologists will need to be equipped to understand and manage drivers for drug relapse.^126^

Summary of six processes in perioperative management of patients with OUD

1. Preoperative screening.
2. Patient education/shared decision‐making and expectation setting.
3. Preoperative optimization and plan of care.
4. Multimodal analgesia.
5. Postoperative management.
6. Discharge planning and follow‐up (warm hand‐off).

### Legal clarifications

With the recent passage of the Consolidated Appropriations Act of 2023, there may be some lingering questions about who precisely can prescribe buprenorphine. Previously, an “X‐waiver” was needed to provide a prescription for milligram‐dose buprenorphine when used specifically for treatment of OUD *but not for analgesia*. In addition, previously, 8 h of training (for physicians, 24 hours for advanced practice providers) and ability to provide direct ancillary services was needed to obtain an X‐waiver. However, with the new legislation, clinicians in the United States no longer need an X‐waiver to prescribe buprenorphine in any of its formulations for any indication; only a valid DEA license is needed, just as it would be to prescribe any other controlled substance. There is, however, now a one‐time eight‐hour requirement for DEA renewal per the Medication Access and Training Expansion (MATE) Act.^127^

### Harm reduction strategies and importance of naloxone

All patients with OUD should be addressed in a caring manner with harm reduction strategies in mind. In this context, patients with OUD should be given take‐home naloxone, prior to discharge.^128^ Distribution of naloxone has been associated with decreased overdose mortality in the community.^129^ Additionally, programs can be established to provide patient education on overdose prevention, including not using alone, use of rapid fentanyl test strips, having a naloxone kit nearby, and dangers of using after a period of detoxification or lowered use.^108^ Approximately 75% of overdose deaths occur in the home, so availability of naloxone is an important step in harm reduction.

## SUMMARY

We are faced with the perioperative consequences of the opioid crisis for many years to come. Expansion to access to treatment is greatly needed. All clinicians, regardless of specialty, will be required to receive education in OUD prior to renewing their DEA license in efforts to increase clinicians' ability to recognize, refer, and treat OUD. Anesthesiolgists, who have a knowledge base in analgesia, can provide these services in the perioperative arena. Doing so may provide life‐saving treatment.

## AUTHOR CONTRIBUTIONS

Lynn Kohan drafted and edited the manuscript. Antje Barreveld drafted and edited the manuscript. Sudheer Potru drafted and edited the manuscript. Al Abd‐Elsayd edited the manuscript. Eugene Viscusi drafted and edited the manuscript.

## CONFLICT OF INTEREST STATEMENT

Authors do not have COI that is relevant to this article. Dr. Abd‐Elsayed is an Editorial Board member of Pain Practice and a co‐author of this article. Dr. Antje Barreveld is an advisor for Lin Health and Consultant and speaker for Vertex Pharmaceuticals, Inc. To minimize bias, they were excluded from all editorial decision‐making related to the acceptance of this article for publication.

## PATIENT CONSENT STATEMENT

Patient consent is not applicable to this manuscript.

## Data Availability

Data sharing not applicable to this article as no datasets were generated or analyzed during the current study.

## References

[1] Keyes KM , Rutherford C , Hamilton A , Barocas JA , Gelberg KH , Mueller PP , et al. What is the prevalence of and trend in opioid use disorder in the United States from 2010 to 2019? Using multiplier approaches to estimate prevalence for an unknown population size. Drug Alcohol Depend Rep. 2022;3:100052. 35783994 10.1016/j.dadr.2022.100052 PMC9248998

[2] U.S. Opioid Dispensing Rate Maps | Drug Overdose | CDC Injury Center [accessed 2022 Dec 4]. Available from: https://www.cdc.gov/drugoverdose/rxrate‐maps/index.html

[3] CDC, National Center for Health Statistics, Office of Communication . CDC press release November 17, 2021: drug overdose deaths in the U.S. top 100,000 annually (301)458‐4800. Available from: https://www.cdc.gov/nchs/nvss/vsrr/drug‐overdose‐data.htm

[4] Comerci G Jr , Katzman J , Duhigg D . Controlling the swing of the opioid pendulum. N Engl J Med. 2018;378(8):691–693. 29466151 10.1056/NEJMp1713159

[5] Coffin PO , Barreveld AM . Inherited patients taking opioids for chronic pain – considerations for primary care. N Engl J Med. 2022;386(7):611–613. 35148038 10.1056/NEJMp2115244 PMC9555806

[6] Dowell D , Ragan KR , Jones CM , Baldwin GT , Chou R . CDC clinical practice guideline for prescribing opioids for pain – United States, 2022. MMWR Recomm Rep. 2022;71:1–95. 10.15585/mmwr.rr7103a1 PMC9639433 36327391

[7] CMS.gov . NHE fact sheet [accessed 2023 Dec 2]. Available from: https://www.cms.gov/research‐statistics‐data‐and‐systems/statistics‐trends‐and‐reports/nationalhealthexpenddata/nhe‐fact‐sheet.html

[8] Ti L , Ti L . Leaving the hospital against medical advice among people who use illicit drugs: a systematic review. Am J Public Health. 2015;105:e53–e59. 26469651 10.2105/AJPH.2015.302885 PMC4638247

[9] Science Daily. Science News [accessed 2023 Dec 2]. Available from: https://www.sciencedaily.com/releases/2019/08/190820081846.htm

[10] White S , Bird SM , Merrall EL , Hutchinson SJ . Drugs‐related death soon after hospital‐discharge among drug treatment clients. PLoS One. 2015;10(11):e0141073. 26539701 10.1371/journal.pone.0141073 PMC4634860

[11] Wilson JM , Farley KX , Aizpuru M , Wagner ER , Bradbury TL , Guild GN . The impact of preoperative opioid use disorder on complications and costs following primary total hip and knee arthroplasty. Adv Orthop. 2019;2019:9319480. 31929911 10.1155/2019/9319480 PMC6939449

[12] Lui B , Aaronson JA , Tangel V , Quincy M , Weinberg R , Abramovitz SE , et al. Opioid use disorder and maternal outcomes following cesarean delivery: a multistate analysis, 2007–2014. J Comp Eff Res. 2020;9(10):667–677. 32648478 10.2217/cer-2020-0050

[13] Boltunova A , Bailey C , Weinberg R , Ma X , Thalappillil R , Tam CW , et al. Preoperative opioid use disorder is associated with poorer outcomes after coronary bypass and valve surgery: a multistate analysis, 2007–2014. J Cardiothorac Vasc Anesth. 2020;34(12):3267–3274. 32620485 10.1053/j.jvca.2020.06.006

[14] Lee D , Armaghani S , Archer KR , Bible J , Shau D , Kay H , et al. Preoperative opioid use as a predictor of adverse postoperative self‐reported outcomes in patients undergoing spine surgery. J Bone Joint Surg Am. 2014;96(11):e89. 24897746 10.2106/JBJS.M.00865

[15] American Society of Addiction Medicine . Definition of addiction [accessed 2023 Dec 2]. Available from: https://www.asam.org/quality‐care/definition‐of‐addiction

[16] American Psychiatric Association . Diagnostic and statistical manual of mental disorders. Fifth ed. Washington, DC: American Psychiatric Association; 2013. p. 541.

[17] Sordo L , Barrio G , Bravo MJ , Indave BI , Degenhardt L , Wiessing L , et al. Mortality risk during and after opioid substitution treatment: systematic review and meta‐analysis of cohort studies. BMJ. 2017;357:j1550. 28446428 10.1136/bmj.j1550 PMC5421454

[18] Jones CM , McCance‐Katz EF . Co‐occurring substance use and mental disorders among adults with opioid use disorder. Drug Alcohol Depend. 2019;197(1):78–82. 30784952 10.1016/j.drugalcdep.2018.12.030

[19] Blanco C , Iza M , Schwartz RP , Rafful C , Wang S , Olfson MJD . Probability and predictors of treatment‐seeking for prescription opioid use disorders: a national study. Drug Alcohol Depend. 2013;131(1–2):143–148. 23306097 10.1016/j.drugalcdep.2012.12.013 PMC3636152

[20] Wang PS , Berglund P , Olfson M , Pincus HA , Wells KB , Kessler RC . Failure and delay in initial treatment contact after first onset of mental disorders in the national comorbidity survey replication. Arch Gen Psychiatry. 2005;62(6):603–613. 15939838 10.1001/archpsyc.62.6.603

[21] Mackey K , Veazie S , Anderson J , Bourne D , Peterson K . Barriers and facilitators to the use of medications for opioid use disorder: a rapid review. J Gen Intern Med. 2020;35(Suppl 3):954–963. 33145687 10.1007/s11606-020-06257-4 PMC7728943

[22] Barry CL , McGinty EE , Pescosolido BA , Goldman HH . Stigma, discrimination, treatment effectiveness, and policy: public views about drug addiction and mental illness. Psychiatr Serv. 2014;65(10):1269–1272. 25270497 10.1176/appi.ps.201400140 PMC4285770

[23] Brondani MA , Alan R , Donnelly L . Stigma of addiction and mental illness in healthcare: the case of patients' experiences in dental settings. PLoS One. 2017;12(5):e0177388. 28531176 10.1371/journal.pone.0177388 PMC5439661

[24] Livingston JD , Adams E , Jordan M , MacMillan Z , Hering R . Primary care physicians' views about prescribing methadone to treat opioid use disorder. Subst Use Misuse. 2018;53(2):344–353. 28853970 10.1080/10826084.2017.1325376

[25] Kennedy‐Hendricks A , Busch SH , McGinty EE , Bachhuber MA , Niederdeppe J , Gollust SE , et al. Primary care physicians' perspectives on the prescription opioid epidemic. Drug Alcohol Depend. 2016a;165:61–70. 27261154 10.1016/j.drugalcdep.2016.05.010 PMC4939126

[26] McGinty E , Pescosolido B , Kennedy‐Hendricks A , Barry CL . Communication strategies to counter stigma and improve mental illness and substance use disorder policy. Psychiatr Serv. 2017;69(2):136–146. 28967320 10.1176/appi.ps.201700076 PMC5794622

[27] Kelly JF , Westerhoff CM . Does it matter how we refer to individuals with substance‐related conditions? A randomized study of two commonly used terms. Int J Drug Policy. 2010;21(3):202–207. 20005692 10.1016/j.drugpo.2009.10.010

[28] Volkow ND , Frieden TR , Hyde PS , Cha SS . Medication‐assisted therapies—tackling the opioid‐overdose epidemic. N Engl J Med. 2014;370(22):2063–2066. 24758595 10.1056/NEJMp1402780

[29] Monico LB , Gryczynski J , Mitchell SG , Schwartz RP , O'Grady KE , Jaffe JH . Buprenorphine treatment and 12‐step meeting attendance: conflicts, compatibilities, and patient outcomes. J Subst Abus Treat. 2015;57:89–95. 10.1016/j.jsat.2015.05.005 PMC4560966 25986647

[30] American Board of Medical Specialties (ABMS) . ABMS officially recognizes addiction medicine as a subspecialty; 2016 (Press release on march 14, 2016) [accessed 2022 Oct 27]. Avaialable from: https://www.abms.org/news‐events/abms‐officially‐recognizes‐addiction‐medicine‐as‐a‐subspecialty

[31] Allen B , Nolan ML , Paone D . Underutilization of medications to treat opioid disorder: what role does stigma play? Subst Abus. 2019;40(4):459–465. 31550201 10.1080/08897077.2019.1640833

[32] Consolidated Appropriations Act of 2023 . Summary of appropriations provisions by subcommittee [accessed 2023 May 18]. Available from: https://appropriations.house.gov/sites/democrats.appropriations.house.gov/files/FY23%20Summary%20of%20Appropriations%20Provisions.pdf

[33] Fiscella K , Wakeman SE , Beletsky L . Buprenorphine deregulation and mainstreaming treatment for opioid use disorder: X the X waiver. JAMA Psychiatr. 2019;76(3):229–230. 10.1001/jamapsychiatry.2018.3685 30586140

[34] Liebschutz JM , Crooks D , Herman D , Anderson B , Tsui J , Meshesha LZ , et al. Buprenorphine treatment for hospitalized, opioid‐dependent patients: a randomized clinical trial. JAMA Intern Med. 2014;174(8):1369–1376. 25090173 10.1001/jamainternmed.2014.2556 PMC4811188

[35] D'Onofrio G , O'Connor PG , Pantalon MV , Chawarski MC , Busch SH , Owens PH , et al. Emergency department–initiated buprenorphine/naloxone treatment for opioid dependence: a randomized clinical trial. JAMA. 2015;313(16):1636–1644. 25919527 10.1001/jama.2015.3474 PMC4527523

[36] O'Connor PG , Nyquist JG , McLellan AT . Integrating addiction medicine into graduate medical education in primary care: the time has come. Ann Intern Med. 2011;154(1):56–59. 21200039 10.7326/0003-4819-154-1-201101040-00008

[37] John S , Boorman DW , Potru S . Identifying barriers to buprenorphine treatment for patients with opioid use disorder among anesthesiologists and pain practitioners: a survey study. J Addict Med. 2022;e94‐e100. 10.1097/ADM.0000000000001066 36001078

[38] US Department of Justice Drug Enforcement Administration [accessed 2023 Apr 24]. Available from: https://www.deadiversion.usdoj.gov/pubs/docs/MATE_Training_Letter_Final.pdf

[39] SAMHSA . Recommendations for curricular elements in substance use disorders training [accessed 2023 Apr 26]. Available from: https://www.samhsa.gov/medications‐substance‐use‐disorders/provider‐support‐services/recommendations‐curricular‐elements‐substance‐use‐disorders‐training

[40] Suen LW , Makam AN , Snyder HR , Repplinger D , Kushel MB , Martin M , et al. National prevalence of alcohol and other substance use disorders among emergency department visits and hospitalizations: NHAMCS 2014‐2018. J Gen Intern Med. 2022;37(10):2420–2428. 34518978 10.1007/s11606-021-07069-w PMC8436853

[41] Gryczynski J , McNeely J , Wu LT , Subramaniam GA , Svikis DS , Cathers LA , et al. Validation of the TAPS‐1: a four‐item screening tool to identify unhealthy substance use in primary care. J Gen Intern Med. 2017;32(9):990–996. 28550609 10.1007/s11606-017-4079-x PMC5570743

[42] Fernandez A , Waljee J , Gunaseelan V , Brummet C , Englesbe M , Bicket M . Prevalence of unhealhy substance use characteristics among patients presenting for surgery. Ann Surg. 2023;278(4):e740–547. 36538617 10.1097/SLA.0000000000005767 PMC10205913

[43] National Institute on Drug Abuse Screening and Assessment Tools Chart [accessed 2022 Dec 15]. Available from: https://www.drugabuse.gov/nidamed‐medical‐health‐professionals/screening‐tools‐resources/chart‐screening‐tools

[44] Ward EN , Quaye AN , Wilens TE . Opioid use disorders: perioperative management of a special population. Anesth Analg. 2018;127(2):539–547. 29847389 10.1213/ANE.0000000000003477 PMC6523021

[45] Huxtable CA , Roberts LJ , Somogyi AA , MacIntyre PE . Acute pain management in opi‐ oid‐tolerant patients: a growing challenge. Anaesth Intensive Care. 2011;39:804–823. 21970125 10.1177/0310057X1103900505

[46] Mariano ER , Dickerson DM , Szokol JW , Harned M , Mueller JT , Philip BK , et al. A multisociety organizational consensus process to define guiding principles for acute perioperative pain management. Reg Anesth Pain Med. 2022;47(2):118–127. 34552003 10.1136/rapm-2021-103083

[47] Dickerson DM , Mariano ER , Szokol JW , Harned M , Clark RM , Mueller JT , et al. Multiorganizational consensus to define guiding principles for perioperative pain management in patients with chronic pain, preoperative opioid tolerance, or substance use disorder. Reg Anesth Pain Med. 2023;49:725. 10.1136/rapm-2023-104435 37185214

[48] Thiesset HF , Schliep KC , Stokes SM , Valentin VL , Gren LH , Porucznik CA , et al. Opioid misuse and dependence screening practices prior to surgery. J Surg Res. 2020;252:200–205. 32283333 10.1016/j.jss.2020.03.015 PMC8668076

[49] You DS , Mardian AS , Darnall BD , Chen CA , De Bruyne K , Flood PD , et al. A brief screening tool for opioid use disorder: EMPOWER study expert consensus protocol. Front Med (Lausanne). 2021;8:591201. 33869240 10.3389/fmed.2021.591201 PMC8044786

[50] Volpe DA , Tobin GAM , Mellon RD . Uniform assessment and ranking of opioid mu receptor binding constants for selected opioid drugs. Regul Toxicol Pharmacol. 2011;59:385–390. 21215785 10.1016/j.yrtph.2010.12.007

[51] Coe MA , Lofwall MR , Walsh SL . Buprenorphine pharmacology review: update on transmucosal and long‐acting formulations. J Addict Med. 2019;13(2):93–103. 30531584 10.1097/ADM.0000000000000457 PMC7442141

[52] Gowing L , Ali R , White JM , Mbewe D , Cochrane Drugs and Alcohol Group . Buprenorphine for managing opioid withdrawal. Cochrane Database Syst Rev. 2017;2:CD002025. 28220474 10.1002/14651858.CD002025.pub5 PMC6464315

[53] Urnoski E . Why is buprenorphine coformulated with naloxone? JAAPA. 2017;30(11):44–45. 10.1097/01.JAA.0000525919.14882.ed 29064938

[54] Kornfeld H , Manfredi L . Effectiveness of full agonist opioids in patients stabilized on buprenorphine undergoing major surgery: a case series. Am J Ther. 2010;17(5):523–528. 19918165 10.1097/MJT.0b013e3181be0804

[55] US National Library of Medicine . Buprenorphine. DailyMed; 2020 [accessed 2022 Sep 15]. Available from: https://dailymed.nlm.nih.gov/dailymed/search.cfm?labeltype=all&query=buprenorphine

[56] Greenwald M , Johanson CE , Bueller J , Chang Y , Moody DE , Kilbourn M , et al. Buprenorphine duration of action: mu‐opioid receptor availability and pharmacokinetic and behavioral indices. Biol Psychiatry. 2007;61(1):101–110. 16950210 10.1016/j.biopsych.2006.04.043

[57] Quaye AN , Zhang Y . Perioperative management of buprenorphine: solving the conundrum. Pain Med. 2018;20(7):1395–1408. 10.1093/pm/pny217 PMC7963209 30500943

[58] Kohan L , Potru S , Barreveld AM , Sprintz M , Lane O , Aryal A , et al. Buprenorphine management in the perioperative period: educational review and recommendations from a multisociety expert panel. Reg Anesth Pain Med. 2021;46(10):840–859. 34385292 10.1136/rapm-2021-103007

[59] Anderson TA , Quaye ANA , Ward EN , Wilens TE , Hilliard PE , Brummett CM . To stop or not, that is the question. Anesthesiology. 2017;126(6):1180–1186. 28511196 10.1097/ALN.0000000000001633 PMC7041233

[60] Childers JW , Arnold RM . Treatment of pain in patients taking buprenorphine for opioid addiction # 221. J Palliat Med. 2012;15(5):613–614. 22577788 10.1089/jpm.2012.9591

[61] Jonan A , Kaye A , Urman R . Buprenorphine formulations: clinical best practice strategies recommendations for perioperative management of patients undergoing surgical or interventional pain procedures. Pain Physician. 2018;21(1):E1–E12. 29357325

[62] Sen S , Arulkumar S , Cornett EM , Gayle JA , Flower RR , Fox CJ , et al. New pain management options for the surgical patient on methadone and buprenorphine. Curr Pain Headache Rep. 2016;20(3):16. 26879874 10.1007/s11916-016-0549-9

[63] Alford D , Compton P , Samet J . Acute pain management for patients receiving maintenance methadone or buprenorphine therapy. Ann Intern Med. 2007;48:127–134. 10.7326/0003-4819-144-2-200601170-00010 PMC1892816 16418412

[64] Acampora GA , Nisavic M , Zhang Y . Perioperative buprenorphine continuous maintenance and administration simultaneous with full opioid agonist: patient priority at the interface between medical disciplines. J Clin Psychiatry. 2020;81(1):19. 10.4088/JCP.19com12810 31917908

[65] Wyse JJ , Herreid‐O'Neill A , Dougherty J , Shull S , Mackey K , Priest KC , et al. Perioperative management of buprenorphine/naloxone in a large, national health care system: a retrospective cohort study. J Gen Intern Med. 2022;37(12):2998–3004. 34545469 10.1007/s11606-021-07118-4 PMC9485300

[66] Macintyre PE , Russell RA , Usher KAN , Gaughwin M , Huxtable CA . Pain relief and opioid requirements in the first 24 hours after surgery in patients taking buprenorphine and methadone opioid substitution therapy. Anaesth Intensive Care. 2013;41(2):222–230. 23530789 10.1177/0310057X1304100212

[67] Schuster B , Bell B , Massoll A , White S . Continuation versus discontinuation of buprenorphine in the perioperative setting: a retrospective study. Cureus. 2022;14(3):e23385. 35481308 10.7759/cureus.23385 PMC9033510

[68] Hansen LE , Stone GL , Matson CA , Tybor DJ , Pevear ME , Smith EL . Total joint arthroplasty in patients taking methadone or buprenorphine/naloxone preoperatively for prior heroin addiction: a prospective matched cohort study. J Arthroplast. 2016;31:1698–1701. 10.1016/j.arth.2016.01.032 26899477

[69] Höflich AS , Langer M , Jagsch R , Bäwert A , Winklbaur B , Fischer G , et al. Peripartum pain management in opioid dependent women. Eur J Pain. 2012;16:574–584. 22396085 10.1016/j.ejpain.2011.08.008 PMC3290684

[70] Vilkins AL , Bagley SM , Hahn KA , Rojas‐Miguez F , Wachman EM , Saia K , et al. Comparison of post‐cesarean section opioid analgesic requirements in women with opioid use disorder treated with methadone or buprenorphine. J Addict Med. 2017;11:397–401. 28727661 10.1097/ADM.0000000000000339

[71] Silva MJ , Rubinstein A . Continuous perioperative sublingual buprenorphine. J Pain Palliat Care Pharmacother. 2016;30(4):289–293. 27736284 10.1080/15360288.2016.1231734

[72] Substance Abuse Mental Health Services Administration (SAMHSA) . Medications for opioid use disorder: for healthcare and addiction professionals, policymakers, patients and families. Updated 2020. Published online 2020. Available from: https://store.samhsa.gov/sites/default/files/SAMHSA_Digital_Download/PEP20‐02‐01‐006_508.pdf 34928548

[73] Bentzley BS , Barth KS , Back SE , Book SW . Discontinuation of buprenorphine maintenance therapy: perspectives and outcomes. J Subst Abus Treat. 2015;52:48–57. 10.1016/j.jsat.2014.12.011 PMC4382404 25601365

[74] Mehta D , Thomas V , Johnson J , Brooke S , Cortina S , Berger L . Continuation of buprenorphine to facilitate postoperative pain management for patients on buprenorphine opioid agonist therapy. Pain Physician. 2020;23:E163–E174. 32214293

[75] Weiss RD , Potter JS , Fiellin DA , Byrne M , Connery HS , Dickinson W , et al. Adjunctive counseling during brief and extended buprenorphine‐naloxone treatment for prescription opioid dependence: a 2‐phase randomized controlled trial. Arch Gen Psychiatry. 2011;68(12):1238–1246. 22065255 10.1001/archgenpsychiatry.2011.121 PMC3470422

[76] SAMHSA . Medications for opioid use disorder. Treatment Improvement Protocol (TIP) Series 63, Full Document. HHS Publication No. (SMA) 19‐5063FULLDOC. Rockville, MD; 2018.

[77] Scholzen E , Zeng AM , Schroeder KM . Perioperative management and analgesia for patients taking buprenorphine and other forms of medication‐assisted treatment for substance abuse disorders. Adv Anesth. 2019;37:65–86. 31677660 10.1016/j.aan.2019.08.002

[78] Quaye AN , Zhang Y . Perioperative management of buprenorphine: solving the conundrum. Pain Med. 2018;20(7):1–14. 10.1093/pm/pny217 PMC7963209 30500943

[79] Veazie S , Mackey K , Bourne MD , Peterson K . Evidence brief: managing acute pain in patients with opioid use disorder on medication‐assisted treatment supplementary materials. Published Online 2019. Available from: https://www.ncbi.nlm.nih.gov/books/NBK549201/ 31670924

[80] Warltier DC , Mitra S , Sinatra RS . Perioperative management of acute pain in the opioid‐dependent patient. Anesthesiology. 2004;101:212–227. 15220793 10.1097/00000542-200407000-00032

[81] Buresh M , Ratner J , Zgierska A , Gordin V , Alvanzo A . Treating perioperative and acute pain in patients on buprenorphine: narrative literature review and practice recommendations. J Gen Intern Med. 2020;35(12):3635–3643. 32827109 10.1007/s11606-020-06115-3 PMC7728902

[82] Larach DB , Hah JM , Brummett CM . Perioperative opioids, the opioid crisis, and the anesthesiologist. Anesthesiology. 2022;136:594–608. 35108351 10.1097/ALN.0000000000004109 PMC8904272

[83] Goel A , Azargive S , Weissman JS , Shanthanna H , Hanlon JG , Samman B , et al. Perioperative pain and addiction interdisciplinary network (PAIN) clinical practice advisory for perioperative management of buprenorphine: results of a modified Delphi process. Br J Anaesth. 2019;123(2):e333–e342. 31153631 10.1016/j.bja.2019.03.044 PMC6676043

[84] Lembke A , Ottestad E , Schmiesing C . Patients maintained on buprenorphine for opioid use disorder should continue buprenorphine through the perioperative period. Pain Med. 2019;20(3):425–428. 29452378 10.1093/pm/pny019 PMC6387981

[85] Buprenorphine: an alternative to methadone. Med Lett Drugs Ther. 2003;45:13–15. 12592214

[86] Johnson RE , Fudala PJ , Payne R . Buprenorphine: considerations for pain management. J Pain Symptom Manag. 2005;29:297–326. 10.1016/j.jpainsymman.2004.07.005 15781180

[87] Fishman SM , Wilsey B , Mahajan G , Molina P . Methadone reincarnated: novel clinical applications with related concerns. Pain Med. 2002;3:339–348. 15099239 10.1046/j.1526-4637.2002.02047.x

[88] Walsh SL , Eissenberg T . The clinical pharmacology of buprenorphine: extrapolating from the laboratory to the clinic. Drug Alcohol Depend. 2003;70:S13–S27. 10.1016/s0376-8716(03)00056-5 12738347

[89] Roberts DM , Meyer‐Witting M . High‐dose buprenorphine: perioperative precautions and management strategies. Anaesth Intensive Care. 2005;33:17–25. 15957687 10.1177/0310057X0503300104

[90] Chou R , Gordon DB , de Leon‐Casasola OA , Rosenberg JM , Bickler S , Brennan T , et al. Management of postoperative pain: a clinical practice guideline from the American Pain Society, the American Society of Regional Anesthesia and Pain Medicine, and the American Society of Anesthesiologists' committee on regional anesthesia, executive committee, and administrative council. J Pain. 2016;17(2):131–157. 26827847 10.1016/j.jpain.2015.12.008

[91] Collett BJ . Opioid tolerance: the clinical perspective. Br J Anaesth. 1998;81:58–68. 9771273 10.1093/bja/81.1.58

[92] Mao J , Price DD , Mayer DJ . Mechanisms of hyperalgesia and morphine tolerance: a current view of their possible interactions. Pain. 1995;62:259–274. 8657426 10.1016/0304-3959(95)00073-2

[93] Kehlet H , Dahl JB . The value of “multimodal” or “balanced analgesia” in postoperative pain treatment. Anesth Analg. 1993;77:1048–1056. 8105724 10.1213/00000539-199311000-00030

[94] Botney M , Fields HL . Amitriptyline potentiates morphine analgesia by a direct action on the central nervous system. Ann Neurol. 1983;13:160–164. 6219612 10.1002/ana.410130209

[95] Mitra S , Sinatra RS . Perioperative management of acute pain in the opioid‐dependent patient. Anesthesiology. 2004;101:212–227. 15220793 10.1097/00000542-200407000-00032

[96] Karasz A , Zallman L , Berg K , Gourevitch M , Selwyn P , Arnsten JH , et al. The experience of chronic severe pain in patients undergoing methadone maintenance treatment. J Pain Symptom Manag. 2004;28:517–525. 10.1016/j.jpainsymman.2004.02.025 15504628

[97] Book SW , Myrick H , Malcolm R , Strain EC . Buprenorphine for postoperative pain following general surgery in a buprenorphine‐maintained patient. Am J Psychiatry. 2007;164:979. 10.1176/ajp.2007.164.6.979 17541066

[98] Thakarar K , Weinstein ZM , Walley AY . Optimising health and safety of people who inject drugs during transition from acute to outpatient care: narrative review with clinical checklist. Postgrad Med J. 2016;92(1088):356–363; and Harm Reduction Coalition. A Safety Manual for Injection Drug Users. [accessed 2023 Apr 26]. Available from: https://harmreduction.org/wp‐content/uploads/2011/12/getting‐off‐ right.pdf 27004476 10.1136/postgradmedj-2015-133720 PMC4967553

[99] NIDA . Effective treatments for opioid addiction; 2016 [accessed 2020 Oct 2]. Available from: https://wwwdrugabusegov/publications/effective‐treatments‐opioid‐addiction

[100] Hickman M , Steer C , Tilling K , Lim AG , Marsden J , Millar T , et al. The impact of buprenorphine and methadone on mortality: a primary care cohort study in the United Kingdom. Addiction. 2018;113(8):1461–1476. 29672985 10.1111/add.14188 PMC6282737

[101] Love JS , Perrone J , Nelson LS . Should buprenorphine be administered to patients with opioid withdrawal in the emergency department? Ann Emerg Med. 2018;72(1):26–28. 29103795 10.1016/j.annemergmed.2017.10.002

[102] Mattick RP , Breen C , Kimber J , Davoli M . Buprenorphine maintenance versus placebo or methadone maintenance for opioid dependence. Cochrane Database Syst Rev. 2014;(2):CD002207. 24500948 10.1002/14651858.CD002207.pub4 PMC10617756

[103] Poorman E . The number needed to prescribe – what would it take to expand access to buprenorphine? NEJM. 2021;384(19):1783–1784. 33983689 10.1056/NEJMp2101298

[104] Velez CM , Nicolaidis C , Korthuis PT , Englander H . “It's been an experience, a life learning experience”: a qualitative study of hospitalized patients with substance use disorders. J Gen Intern Med. 2017;32(3):296–303. 27957661 10.1007/s11606-016-3919-4 PMC5331007

[105] Englander H , Weimer M , Solotaroff R , Nicolaidis C , Chan B , Velez C , et al. Planning and designing the improving addiction care team (IMPACT) for hospitalized adults with substance use disorder. J Hosp Med. 2017;12(5):339–342. 28459904 10.12788/jhm.2736 PMC5542562

[106] Stein MD , O'Sullivan PS , Ellis P , Perrin H , Wartenberg A . Utilization of medical services by drug abusers in detoxification. J Subst Abus. 1993;5:187–193. 10.1016/0899-3289(93)90062-g 8104569

[107] Patel N , Schwenk ES , Ferd P , Torjman MC , Baratta JL , Viscusi ER . An anesthesiologist‐led inpatient buprenorphine initiative. Pain Pract. 2021;21(6):692–697. 33484230 10.1111/papr.12996

[108] Herscher M , Fine M , Navalurkar R , Hirt L , Wang L . Diagnosis and management of opioid use disorder in hospitalized patients. Med Clin North Am. 2020;104(4):695–708. 32505261 10.1016/j.mcna.2020.03.003

[109] Shanahan CW , Beers D , Alford DP , Brigandi E , Samet JH . A transitional opioid program to engage hospitalized drug users. J Gen Intern Med. 2010;25(8):803–808. 20237960 10.1007/s11606-010-1311-3 PMC2896583

[110] Chen KY , Chen L , Mao J . Buprenorphine‐naloxone therapy in pain management. Anesthesiology. 2014;120:1262–1274. 24509068 10.1097/ALN.0000000000000170 PMC3999180

[111] Suzuki J , DeVido J , Kalra I , Mittal L , Shah S , Zinser J , et al. Initiating buprenorphine treatment for hospitalized patients with opioid dependence: a case series. Am J Addict. 2015;24:10–14. 25823630 10.1111/ajad.12161

[112] Weimer MB , Guerra M , Morrow G , Adams K . Hospital‐based buprenorphine micro‐dose initiation. J Addict Med. 2021;15(3):255–257. 32960820 10.1097/ADM.0000000000000745

[113] Button D , Hartley J , Robbins J , Levander XA , Smith NJ , Englander H . Low‐dose buprenorphine initiation in hospitalized adults with opioid use disorder: a retrospective cohort analysis. J Addict Med. 2022;16(2):e105–e111. 34001775 10.1097/ADM.0000000000000864 PMC8595358

[114] Klaire S , Zivanovic R , Barbic SP , Sandhu R , Mathew N , Azar P . Rapid micro‐induction of buprenorphine/naloxone for opioid use disorder in an inpatient setting: a case series. Am J Addict. 2019;28(4):262–265. 30901127 10.1111/ajad.12869

[115] Thakrar AP , Jablonski L , Ratner J , Rastegar DA . Micro‐dosing intravenous buprenorphine to rapidly transition from full opioid agonists. J Addict Med. 2022;16(1):122–124. 33758112 10.1097/ADM.0000000000000838

[116] Herring AA , Vosooghi AA , Luftig J , Anderson ES , Zhao X , Dziura J , et al. High‐dose buprenorphine induction in the emergency Department for Treatment of opioid use disorder. JAMA Netw Open. 2021;4(7):e2117128. 34264326 10.1001/jamanetworkopen.2021.17128 PMC8283555

[117] D'Onofrio G , Edelman EJ , Hawk KF , Pantalon MV , Chawarski MC , Owens PH , et al. Implementation facilitation to promote emergency department‐initiated buprenorphine for opioid use disorder: protocol for a hybrid type III effectiveness‐implementation study (project ED HEALTH). Implement Sci. 2019;14(1):48. 31064390 10.1186/s13012-019-0891-5 PMC6505286

[118] Solomon KT , O'Connor J , Gibbons JB , Kilaru AS , Feder KA , Xue L , et al. Association between hospital adoption of an emergency department treatment pathway for opioid use disorder and patient initiation of buprenorphine after discharge. JAMA Health Forum. 2023;4(3):e230245. 36961457 10.1001/jamahealthforum.2023.0245 PMC10313142

[119] Regan S , Howard S , Powell E , Martin A , Dutta S , Hayes BD , et al. Emergency department‐initiated buprenorphine and referral to follow‐up addiction care: a program description. J Addict Med. 2022;16(2):216–222. 34145185 10.1097/ADM.0000000000000875

[120] Snyder H , Chau B , Kalmin MM , Speener M , Campbell A , Moulin A , et al. High‐dose buprenorphine initiation in the emergency department among patients using fentanyl and other opioids. JAMA Netw Open. 2023;6(3):e231572. 36867410 10.1001/jamanetworkopen.2023.1572 PMC9984967

[121] Khatri UG , Lee K , Lin T , D'Orazio JL , Patel MS , Shofer FS , et al. A brief educational intervention to increase ED initiation of buprenorphine for opioid use disorder (OUD). J Med Toxicol. 2022;18(3):205–213. 35415804 10.1007/s13181-022-00890-7 PMC9004452

[122] Hern HG , Goldstein D , Kalmin M , Kidane S , Shoptaw S , Tzvieli O , et al. Prehospital initiation of buprenorphine treatment for opioid use disorder by paramedics. Prehosp Emerg Care. 2022;26(6):811–817. 34505820 10.1080/10903127.2021.1977440

[123] Hern HG , Lara V , Goldstein D , Kalmin M , Kidane S , Shoptaw S , et al. Prehospital buprenorphine treatment for opioid use disorder by paramedics: first year results of the EMS buprenorphine use pilot. Prehosp Emerg Care. 2023;27(3):334–342. 35420925 10.1080/10903127.2022.2061661

[124] Seol A , Chan J , Micham B , Ye Y , Mariano ER , Harrison TK , et al. Acute pain service reduces barriers to buprenorphine/naloxone initiation by using regional anesthesia techniques. Reg Anesth Pain Med. 2023;48:425–427. 36792313 10.1136/rapm-2022-104317

[125] Wesson DR , Ling W . The clinical opiate withdrawal scale (COWS). J Psychoactive Drugs. 2003;35(2):253–259. 12924748 10.1080/02791072.2003.10400007

[126] Myers J , Compton P . Addressing the potential for perioperative relapse in those recovering from opioid use disorder. Pain Med. 2018;19(10):1908–1915. 29186526 10.1093/pm/pnx277

[127] Congress.gov [accessed 2023 Apr 26]. Available from: https://www.congress.gov/bill/117th‐congress/house‐bill/2067?q=%7B%22search%22%3A%5B%22MATE+Act+2021%22%2C%22MATE%22%2C%22Act%22%2C%222021%22%5D%7D&s=1&r=1

[128] Office of the Surgeon General, Assistant Secretary for Health (ASH) . U.S. Surgeon General's Advisory on Naloxone and Opioid Overdose. HHS.gov [accessed 2022 Oct 27]. Available from: https://www.hhs.gov/surgeongeneral/priorities/opioids‐and‐addiction/naloxone‐advisory/index.html

[129] Walley AY , Xuan Z , Hackman HH , Quinn E , Doe‐Simkins M , Sorensen‐Alawad A , et al. Opioid overdose rates and implementation of overdose education and nasal naloxone distribution in Massachusetts: interrupted time series analysis. BMJ. 2013;346:f174. 23372174 10.1136/bmj.f174 PMC4688551

[130] Farrell M . Opiate withdrawal. Addiction. 1994;89(11):1471–1475. 7841858 10.1111/j.1360-0443.1994.tb03745.x

[131] Evidence supporting the effectiveness of an SBIRT [accessed 2022 Dec 2]. Available from: https://www.samhsa.gov/sites/default/files/sbirtwhitepaper_0.pdf

[132] Committee on Standards and Practice Parameters , Apfelbaum JL , Connis RT , Nickinovich DG , American Society of Anesthesiologists Task Force on Preanesthesia Evaluation , Pasternak LR , et al. Practice advisory for preanesthesia evaluation: an updated report by the American Society of Anesthesiologists Task Force on Preanesthesia evaluation. Anesthesiology. 2012;116(3):522–538. 22273990 10.1097/ALN.0b013e31823c1067

[133] Barreveld AM , Mendelson A , Deiling B , Armstrong CA , Viscusi ER , Kohan LR . Caring for our patients with opioid use disorder in the perioperative period: a guide for the anesthesiologist. Anesth Analg. 2023;137(3):488–881. 37590794 10.1213/ANE.0000000000006280

